# Species-specific pharmacology of Trichloro(sulfanyl)ethyl benzamides as transient receptor potential ankyrin 1 (TRPA1) antagonists

**DOI:** 10.1186/1744-8069-3-39

**Published:** 2007-12-17

**Authors:** Lana Klionsky, Rami Tamir, BaoXi Gao, Weiya Wang, David C Immke, Nobuko Nishimura, Narender R Gavva

**Affiliations:** 1 Department of Neuroscience, Amgen, Inc, Thousand Oaks, California, USA; 2 Small Molecule Chemistry, Amgen, Inc, Thousand Oaks, California, USA

## Abstract

Agonists of TRPA1 such as mustard oil and its key component AITC cause pain and neurogenic inflammation in humans and pain behavior in rodents. TRPA1 is activated by numerous reactive compounds making it a sensor for reactive compounds in the body. Failure of AITC, formalin and other reactive compounds to trigger pain behavior in TRPA1 knockout mice, as well as the ability of TRPA1 antisense to alleviate cold hyperalgesia after spinal nerve ligation, suggest that TRPA1 is a potential target for novel analgesic agents. Here, we have characterized CHO cells expressing human and rat TRPA1 driven by an inducible promoter. As reported previously, both human and rat TRPA1 are activated by AITC and inhibited by ruthenium red. We have also characterized noxious cold response of these cell lines and show that noxious cold activates both human and rat TRPA1. Further, we have used CHO cells expressing human TRPA1 to screen a small molecule compound library and discovered that 'trichloro(sulfanyl)ethyl benzamides' (AMG2504, AMG5445, AMG7160 and AMG9090) act as potent antagonists of human TRPA1 activated by AITC and noxious cold. However, trichloro(sulfanyl)ethyl benzamides' (TCEB compounds) displayed differential pharmacology at rat TRPA1. AMG2504 and AMG7160 marginally inhibited rat TRPA1 activation by AITC, whereas AMG5445 and AMG9090 acted as partial agonists. In summary, we conclude that both human and rat TRPA1 channels show similar AITC and noxious cold activation profiles, but TCEB compounds display species-specific differential pharmacology at TRPA1.

## Background

The plant irritant materials such as mustard oil and wasabi are known to cause rapid intense burning sensation [1-3]. Mustard oil causes pain in humans and pain behavior in rodents by excitation of sensory nerve fibers in part due to neurogenic inflammation through release of neuropeptides such as substance P and CGRP and other transmitters from activated nerve endings [3]. The active ingredient in mustard oil, allyl isothiocyanate (AITC) selectively activates a non-selective cation channel, transient receptor potential ankyrin 1 (TRPA1) expressed in the small neurons of the dorsal root and trigeminal ganglia [4,5]. Interestingly, other plant irritant compounds such as allicin from garlic and cinnamaldehyde from cinnamon also activate TRPA1 [5-7]. Since these compounds are capable of forming covalent adducts with thiols, other reactive compounds such as acrolein, iodo-acetamide, N-methylmaleimide, and several others were evaluated and shown to activate TRPA1 through reversible covalent modification of cystenies in the intracellular loops of TRPA1 [8-11]. These studies resulted in the proposal that TRPA1 acts as a sensor for reactive chemicals in the body [12,13]. In agreement with this hypothesis, recently, it was reported that 4-hydroxynonenal, an endogenous aldehyde causes pain and neurogenic inflammation through activation of TRPA1 [14].

In addition to reactive chemical activators, mechanical stimuli and noxious cold have been shown to activate TRPV1 in recombinant expression systems [15,16]. Reactive chemicals such as AITC did not cause pain behavior in TRPA1 knockout mice, unequivocally confirming that their actions are mediated exclusively by TRPA1 [9,17]. On the other hand, noxious cold effect in TRPA1 knockout mice from two different labs differed [9,17,18], questioning the validity of noxious cold activation of TRPA1. However, recent studies clearly showed that noxious cold indeed activates TRPA1 in calcium imaging experiments as well as in single channel recordings [19].

**Figure 1:**
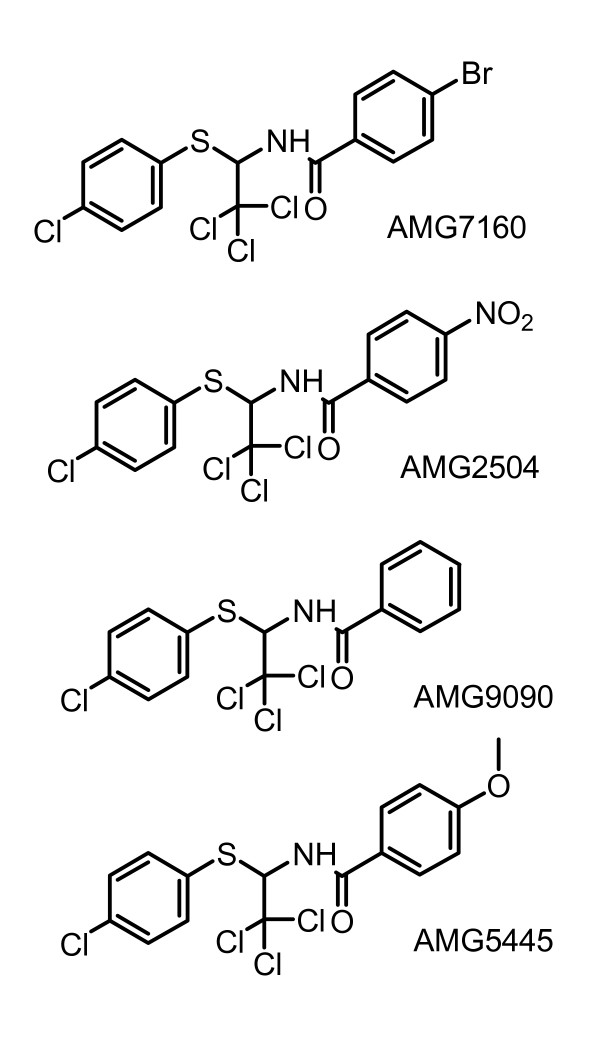
Chemical structures of compounds used in these studies.

Formalin model is widely used to assess pain and to evaluate analgesic drugs in rodents. Recently, formalin was reported to directly activate TRPA1 and mediate the formalin-induced pain behaviors [20]. Both Phase I and Phase II pain behaviors were attenuated in TRPA1 knockout mice. In addition, TRPA1 expression induced in sensory neurons was reported to contribute to cold hyperalgesia after inflammation and nerve injury [21], and antisense knock down of TRPA1 reported to alleviate cold hyperalgesia after spinal nerve ligation in rats [22]. In all, these studies suggest that TRPA1 is a target to identify potential novel analgesics. In our attempts to discover the TRPA1 antagonists, we have used CHO cells recombinantly expressing TRPA1 channels to screen a compound library and found that 'trichloro(sulfanyl)ethyl benzamides' (TCEB compounds; Fig. 1) act as potent and selective antagonists of human TRPA1. Here, we report the pharmacological characterization of TCEB compounds effects on chemical ligand and noxious cold activation of human and rat TRPA1.

## Results

### Characterization of CHO cells expressing human and rat TRPA1

**Figure 2:**
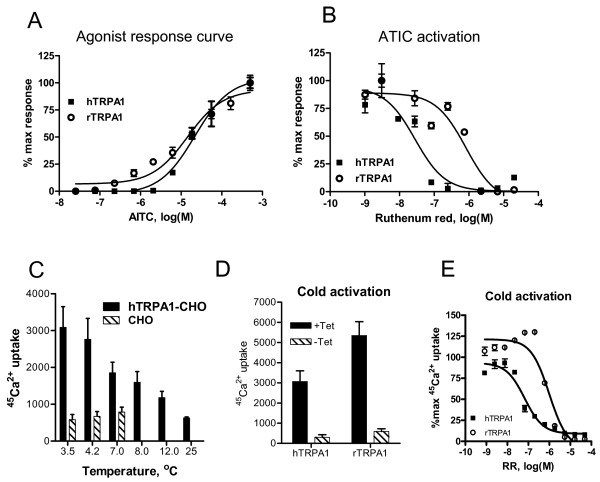
Characterization of CHO cell lines stably expressing human and rat TRPA1. FLASH luminometer was used to measure **(A) **concentration-dependent AITC-induced increase in intracellular calcium, and **(B) **concentration-dependent inhibition of AITC (80 μM) activation by ruthenium red in CHO cells expressing human and rat TRPA1. Each point in the graph is an average ± SEM of an experiment conducted in triplicate. Maximum response of AITC (80 μM) was normalized to 100%. Cold activation profiles of CHO cells expressing human and rat TRPA1 were characterized utilizing cold-induced ^45^Ca^2+ ^uptake assay. **(C) **^45^Ca^2+ ^uptake by CHO cells and CHO cells transfected with human TRPA1 in response to stimulation with temperatures between 3.5 and 25°C. **(D) **Cold temperature (4°C) activation of un-induced and tetracycline-induced CHO cells transfected with either human or rat TRPA1. **(E) **Concentration-dependent inhibition of cold (4°C) activation by ruthenium red in CHO cells expressing human and rat TRPA1. Each point in the graph is an average ± SEM of an experiment conducted in duplicate.

To identify novel TRPA1 antagonists we have established high throughput luminescence readout based functional assays utilizing stable CHO cell lines expressing aequorin cDNA under control of constitutively active promoter and human or rat TRPA1 cDNAs under control of tetracycline inducible promoter. This enabled ad hoc expression of TRPA1 channels for cell based assays without the potential toxic effects of constitutive expression of TRPA1 during freezing and thawing of the cells. To characterize our cell lines we began by testing their functional activity in luminescence based Ca^2+ ^influx assay. Addition of TRPA1 agonist AITC to the cells increased luminescence signal in a concentration-dependent manner (Fig. 2A). EC_50 _values for AITC activation of human and rat TRPA1 channels were 20 ± 5 and 14 ± 3 μM respectively. Based on these results we selected 80 μM AITC to be used for activation of TRPA1 in all antagonist experiments. We then examined the ability of a pore blocker, ruthenium red, to inhibit AITC activation (Fig. 2B). Ruthenium red inhibited AITC activation of both human and rat TRPA1 with IC_50 _values of 29 ± 6 and 937 ± 233 nM, respectively.

Since our automated FLASH luminometer was not equipped for noxious cold activation assays, we employed noxious cold-induced ^45^Ca^2+ ^uptake to monitor TRPA1 activation. First, we evaluated human TRPA1 temperature activation profile (Fig. 2C). We compared ^45^Ca^2+ ^uptake in CHO cells expressing human TRPA1 and in untransfected CHO cells exposed to temperatures between 3.5 and 25°C. Maximum response was observed in TRPA1 expressing CHO cells incubated at 3.5–4.2°C. Minimal induction of ^45^Ca^2+ ^uptake by untrasfected CHO cells was observed at noxious cold (3–5 to 7°C), however cells expressing TRPA1 showed a four to five-fold higher activation at the same temperatures in this assay (Fig. 2C). Further more, there was an apparent temperature drop dependent increase in ^45^Ca^2+ ^uptake in CHO expressing cells expressing human TRPA1. In addition, we also compared response of tetracycline induced versus un-induced human and rat TRPA1 transfected CHO cells to noxious cold in ^45^Ca^2+ ^uptake assay (Fig. 2D). Six to ten-fold increase in ^45^Ca^2+ ^uptake was observed in tetracycline induced cells, suggesting that activation by noxious cold is in fact due to expression of human and rat TRPA1 (Fig. 2D). Next, we evaluated the ability of ruthenium red to inhibit noxious cold activation of TRPA1 (Fig. 2E). Ruthenium red inhibited noxious cold-induced ^45^Ca^2+ ^uptake in CHO cells expressing both human and rat TRPA1 in a concentration-dependent manner with IC_50 _values of 67 ± 4 nM, and 959 ± 26 nM, respectively.

### Trichloro(sulfanyl)ethyl benzamides are potent antagonists of human TRPA1 activated by AITC

In order to screen the small molecule libraries, CHO cells expressing human TRPA1 seeded in 96-well plates were first incubated with 10 μM concentration of individual compounds for one minute to evaluate potential agonism followed by the addition of 80 μM AITC to evaluate antagonist properties for another minute. Compounds that did not display partial agonist activity but exhibited inhibition of AITC-induced calcium influx were characterized further. During this process, we have discovered that four molecules representing trichloro(sulfanyl)ethyl benzamides completely inhibited human TRPA1 activation.

**Figure 3:**
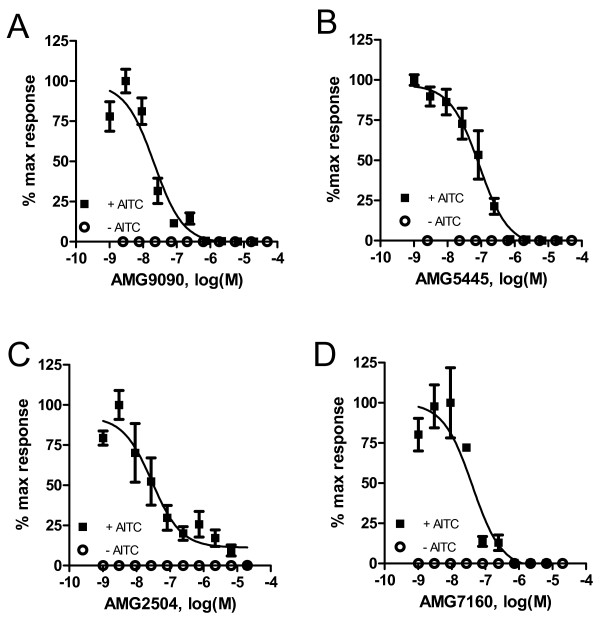
Trichloro(sulfanyl)ethyl benzamides inhibit AITC (80 μM) induced increase in intracellular calcium and inward currents in CHO cells stably expressing human TRPA1 **(A-D)**. Effect of AMG9090 **(A)**, AMG5445 **(B)**, AMG2504 **(C)**, and AMG7160 **(D) **on AITC-induced increase in intracellular calcium in CHO cells expressing human TRPA1 measured in an aequorin-readout assay. Open circles represent the response of cells to the compound itself in the absence of agonist. Each point in the graph is an average ± SEM of an experiment conducted in triplicate.

**Figure 4:**
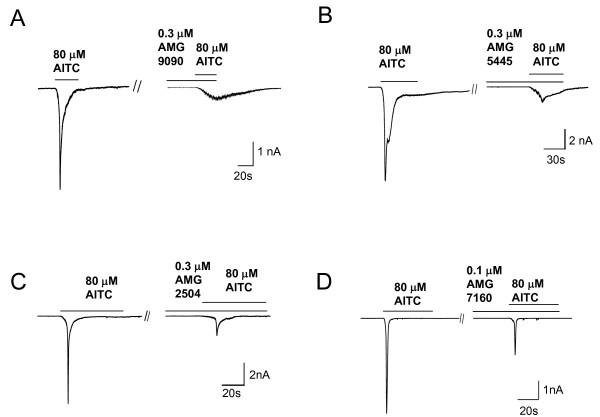
Trichloro(sulfanyl)ethyl benzamides inhibit AITC (80 μM) induced inward currents in CHO cells stably expressing human TRPA1. Representative traces of inward currents evoked by AITC in the absence or presence of AMG9090 **(A)**, AMG5445 **(B)**, AMG2504 **(C)**, and AMG7160 **(D) **are shown. IC_50 _value for each compound was determined from their concentration-dependent inhibition of AITC-induced currents using a PatchXpress 7000A workstation.

Further, we evaluated concentration-dependent effects of TCEB compounds on AITC activation of human TRPA1 in CHO cells (Fig. 3A–D). All four TCEB compounds potently and concentration-dependently inhibited AITC-induced increase in intracellular calcium mediated by TRPA1. The IC_50 _values determined for AMG9090, AMG5445, AMG2504 and AMG7160 were 21 ± 0.6, 91 ± 39, 35 ± 29 and 51 ± 17 nM, respectively. All four TCEB compounds' did not induce any calcium uptake through activation of TRPA1 in these assays, suggesting that these are not partial agonists of TRPA1 (Fig. 3A–D). In addition, we evaluated all four TCEB compounds in electrophysiology, using whole cell voltage-clamp configuration (Fig. 4A–D). In this assay, as predicted, all four TCEB compounds inhibited AITC-induced currents in a concentration-dependent manner with IC_50 _values of 120 ± 31, 260 ± 101, 167 ± 55 and 252 ± 73 nM for AMG9090, AMG5445, AMG2504 and AMG7160, respectively. Two most potent TCEB compounds at inhibiting AITC activation of human TRPA1 in both aequorin based luminescence and electrophysiology assays were AMG9090 and AMG2504.

Our efforts to characterize the nature of TCEB compounds inhibition of AITC activation and to determine the dissociation constants for TCEB compounds were not successful (data not shown). For example, all four TCEB compounds did not shift the concentration response curves of AITC to right, however showed attenuation of maximum response. We believe these results were confounded by the nature of AITC activation of TRPA1, which acts by covalent modification of intracellular cysteines to activate the TRPA1 channels [8].

We have also evaluated the selectivity profile of TCEB compounds among closely related TRP channels. TCEB compounds were found to be selective for TRPA1 among the recombinant TRP family members that we have tested (Table 1). The IC_50 _value for all four TCEB compounds were >20 μM except AMG9090 against capsaicin activated TRPV1, 2-APB activated TRPV3, 4-αPDD activated TRPV4, and icilin activated TRPM8 in cell based assays that measure agonist-induced increases in intracellular calcium in CHO cells recombinantly expressing the appropriate TRP channel. AMG9090 inhibited TRPM8 with an IC_50 _value of 2.43 μM.

**Table 1:** Effects of trichloro(sulfanyl)ethyl benzamides on capsaicin activation of TRPV1, icilin-activated TRPM8, 2-APB-activated TRPV3 and 4α-PDD-induced TRPV4. The ability of compounds to inhibit channel activation was evaluated up to a maximum concentration of 5 μM.

Compound	hTRPV1	hTRPM8	hTRPV3	hTRPV4
AMG9090	>5	2.43	>5	>5
AMG5445	ND	>5	>5	>5
AMG2504	>5	>5	>5	>5
AMG7160	>5	>5	>5	>5

### TCEB compounds are potent antagonists of human TRPA1 activated by noxious cold

**Figure 5:**
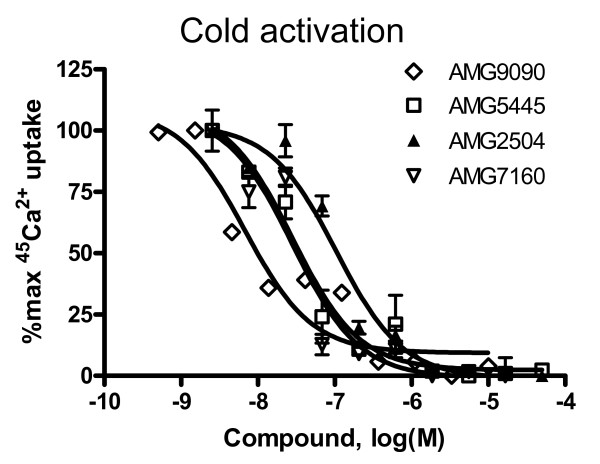
Trichloro(sulfanyl)ethyl benzamides inhibit cold temperature (4°C) induced ^45^Ca^2+ ^uptake in CHO cells stably expressing human TRPA1. Concentration-response curves were generated utilizing ^45^Ca^2+ ^uptake assays as described under *Materials and Methods*. Each point in the graph is an average ± SEM of an experiment conducted in duplicate.

Since noxious cold (3.5 and 4.2°C) induced a significant ^45^Ca^2+ ^uptake into CHO cells in a TRPA1 dependent manner, we evaluated the ability of TCEB compounds to inhibit this response. In this assay, all four TCEB compounds inhibited human TRPA1 activation by 3.5°C temperature. The IC_50 _values determined for AMG9090, AMG5445, AMG2504 and AMG7160 are 7 ± 0.3, 29 ± 6, 105 ± 9 and 28 ± 0.26 nM, respectively (Fig. 5). The rank order of potency for these compounds inhibiting noxious cold activation was AMG9090 > AMG7160 = AMG5445 > AMG2504. In general, it appears that these compounds are more potent at inhibiting noxious cold activation of TRPA1 compared to AITC (compare figures 3 and 5).

### Differential pharmacology of TCEB compounds at rat TRPA1

**Figure 6:**
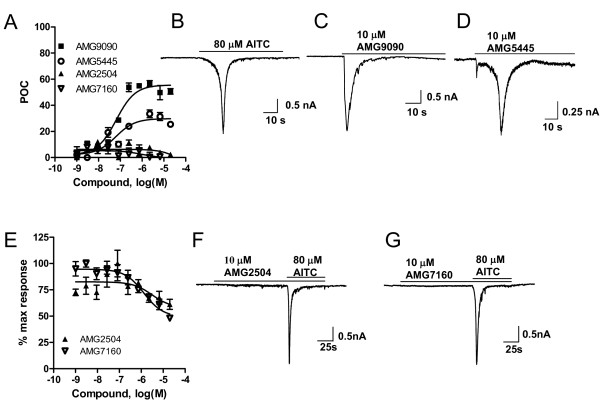
Effect of human TRPA1 antagonists on CHO cells stably expressing rat TRPA1. **(A) **Concentration-response curves generated in aequorin-readout assay Note, AMG9090 and AMG5445 acted as partial agonists. Each point in the graph is presented as percent of AITC response and is an average ± SEM of an experiment conducted in triplicate. Representative traces of inward currents induced by AITC, AMG9090 and AMG5445 are shown in **B**, **C**, and **D **respectively. **(E) **AITC-induced increase in intracellular calcium in CHO cells expressing rat TRPA1 in the absence (100%) or presence of different concentrations of AMG2504 and AMG 7160. Representative current traces induced by AITC in the presence of AMG2504 and AMG 7160 are shown in **F **and **G**, respectively.

Before evaluating TCEB compounds ability to inhibit rat TRPA1 activation, we tested their potential agonism in CHO cells expressing TRPA1. Surprisingly, AMG9090 and AMG5445 induced an increase in intracellular calcium in a concentration dependent manner, suggesting that they are partial agonists at rat TRPA1 (Fig. 6A). Compared to 80 μM AITC efficacy, maximum efficacy of AMG9090 was approximately 50% and EC_50 _value was 66 ± 11 nM. Similarly, maximum efficacy of AMG5445 was approximately 35% with an EC_50 _value of 115 ± 70 nM. In contrast, AMG2504 and AMG7160 did not induce an increase in intracellular calcium up to 50 μM, suggesting that they are not partial agonists (Fig. 6A). We next examined the ability of AMG2504 and AMG7160 to inhibit AITC activation of rat TRPA1. Both compounds showed marginal inhibition of AITC-induced increase in intracellular calcium (IC_50 _value is > 30 μM; Fig. 6E).

We further examined all four TCEB compounds in electrophysiology, using whole cell voltage-clamp configuration. Correlating with agonist-induced aequorin based luminescence read out, both AMG9090 and AMG5445 induced inward currents in CHO cells expressing TRPA1, confirming their partial agonism (Fig. 6C,D). Further, AMG2504 and AMG7160 neither inducing any inward currents by themselves nor significantly blocked currents generated by 80 μM AITC (Fig. 6FG), confirming the observations seen in assays that measured agonist-induced aequorin based luminescence. In summary, TCEB compounds exhibited exactly same differential pharmacology at rat TRPA1 in two different cell based assays.

## Discussion

TRPA1 expression induced in sensory neurons was reported to contribute to cold hyperalgesia after inflammation and nerve injury [21], and antisense knock down of TRPA1 reported to alleviate cold hyperalgesia after spinal nerve ligation in rats [22]. In addition, agonists of TRPA1 cause pain in humans [3] and pain behavior in wild type but not in TRPA1 knockout mice [9,17]. Based on above observations, TRPA1 is considered as a promising target for identification of analgesic drugs. To identify TRPA1 antagonists, first, we generated CHO cells stably integrated with TRPA1 cDNA under a tetracycline inducible expression vector. We then characterized both human and rat TRPA1 expression in CHO cells by measuring AITC-induced increases in intracellular calcium and the ability to inhibit this response by ruthenium red. Although noxious cold activation of TRPA1 is somewhat controversial [4,16], recently it has been proven that noxious cold activates TRPA1 channels characterized by calcium imaging as well as single channel recordings [19]. To evaluate if noxious cold activates TRPA1 channels in our experimental conditions, we employed a radioactive calcium uptake assay in native CHO cells as well as CHO cells that express TRPA1 and confirmed that noxious cold indeed activates both human and rat TRPA1. We have also confirmed noxious cold activation of TRPA1 by evaluating un-induced and tetracycline-induced CHO cells transfected with TRPA1, which showed that noxious cold induces significant ^45^Ca^2+ ^influx only into tetracycline-induced CHO cells transfected with TRPA1. Further, we showed that noxious cold-induced ^45^Ca^2+ ^influx was inhibited by ruthenium red. In summary, these studies confirm that both human and rat TRPA1 are activated by AITC and noxious cold and a pore blocker, ruthenium red inhibits both.

Since we are interested in identifying TRPA1 antagonists, we employed a 96-well format HTS assay to screen compound libraries and identified four TCEB compounds (AMG2504, AMG5445, AMG7160 and AMG9090) as potent antagonists of human TRPA1 activation by AITC. We further confirmed the antagonism of TCEB compounds at human TRPA1 by electrophysiology studies that demonstrated similar IC_50 _values, indicating TCEB compounds are the most potent TRPA1 antagonists reported to date. In addition, all four compounds inhibited noxious cold-activated human TRPA1 channels suggesting that this series of antagonists inhibit two distinct modes of TRPA1 activation. Based on the structures of TCEB compounds, un-substituted phenyl on the amide seems to give greater potency to inhibit AITC activation of human TRPA1. In addition, all three substitutions (4-methoxy phenyl, 4-bromophenyl, and 4-nitrophenyl) at this position did not alter the potency significantly at human TRPA1 channels.

We do not know where these molecules interact within the TRPA1 channel, or if they act as antagonists by modifying the intracellular cysteines so that agonists no longer modify them to activate the channel. Our attempts to determine the dissociation constants for these compounds did not show a clear pattern of whether these compounds are competitive antagonists of AITC. This is further complicated by the fact that AITC activates the TRPA1 channel by intracellular cysteine modifications. Further, it is not known where icilin binds on the TRPA1 channel and what critical residues are required for icilin and noxious cold activation. Chemical ligands are known to interact within the transmembrane domains 2 to 4 region for the other well studied TRP channels such as TRPV1 [23-25], TRPM8 [26]and TRPV4 [27], but critical residues for heat activation are still unknown. In the absence of such details for TRPA1, we can only predict that TCEB series of antagonists probably lock the channel conformation in the closed or non-conducting state so that neither chemical agonists such as AITC nor noxious cold can activate this channel.

Recently, it was reported that calcium directly activates TRPA1 channels [28,29]. It is possible that noxious cold can release intracellular calcium or induce extracellular calcium uptake that might activate TRPA1. In our studies noxious cold did induce uptake of a small amount of extra cellular calcium into CHO cells, however, uptake was four to five-fold higher in TRPA1 expressing cells. Since selective antagonists of TRPA1 potently inhibited noxious cold-induced ^45^Ca^2+ ^uptake into CHO cells expressing TRPA1, we believe that noxious cold does activate TRPA1. Since we cannot completely rule out the contribution of calcium directly activating TRPA1 channels during noxious cold application, it is possible that both calcium and noxious cold might have contributed to TRPA1 activation and concomitant ^45^Ca^2+ ^uptake in our studies. However, TCEB compounds inhibit this ^45^Ca^2+ ^uptake completely confirming that all noxious cold-induced ^45^Ca^2+ ^uptake was TRPA1 mediated.

All four TCEB compounds that we report here inhibited both AITC and noxious cold activation of human TRPA1 but exhibited differential pharmacology at rat TRPA1. AMG9090 and AMG5445 acted as partial agonists whereas AMG2504 and AMG7160 showed a marginal antagonism at rat TRPA1. It is interesting that modifications on the phenyl group on amide on the right hand side do not seem to affect the antagonism of human TRPA1 significantly and by inference the way they interact with the TRPA1 channels to inhibit AITC or cold activation. However, the same modifications dramatically affected the effects of these molecules at rat TRPA1. For example, un-substituted phenyl in AMG9090 and 4-methoxy phenyl in AMG5445 on amide resulted in partial agonism at rat TRPV1 for these two compounds. Furthermore, 4-bromophenyl in AMG7160 and 4-ntrophenyl in AMG2504 on amide did not result in partial agonism but made them almost ineffective against AITC activation of TRPA1. As we mentioned earlier for mechanism(s) of human TRPA1 antagonism, it is unclear how these modifications affect their interactions with rat TRPA1 or how such dramatic effects can arise from their interaction with rat TRPA1. Since we have confirmed the results in two independent readouts (i.e., by assays that measure agonist-induced increase in intracellular calcium and by electrophysiology methods), we believe the observed effects of these compounds at rat TRPA1 are not experimental artifacts.

Recently, significant initial progress has been made toward identifying TRPA1 antagonists that will help evaluate their utility as pain therapeutics. One of these studies demonstrated that TRPA1 mediates the formalin-induced pain, by showing that responses to formalin were attenuated in TRPA1 knockout mice and that pharmacological blockade of TRPA1 by a novel antagonist attenuates formalin-induced pain behaviors in rodents. Further, another TRPA1 antagonist was shown to inhibit CFA-induced mechanical allodynia (Ardem Patapoutian, Spring Pain Conference, 2006, Grand Cayman, BWI), suggesting that TRPA1 might play a role in inflammatory pain. TRPA1 antagonist effects in neuropathic pain models are yet to be determined. Further studies should reveal: i) identification of the binding pocket for non-reactive agonists and antagonists, ii) key residues involved in noxious cold activation, iii) how the antagonist interactions with the TRPA1 affect both chemical ligand and noxious cold activation, and iv) the utility of TRPA1 antagonists as analgesics.

## Methods

The details of cDNA sequences used for generation of stable cell lines were described recently in Gavva et al., 2007. All the cell culture reagents were purchased from Invitrogen (Carlsbad, CA). All compounds used in the study are shown in Figure 1.

### Luminescence readout assay for measuring intracellular calcium

Stable CHO cell lines expressing human TRPA1, TRPM8, TRPV3, TRPV4 and rat TRPA1 were generated using tetracycline inducible T-REx™ expression system from Invitrogen, Inc (Carlsbad, CA). Generation of stable CHO cells expressing human TRPV1 was described previously in Gavva et al., 2005. In order to enable a luminescence readout based on intracellular increase in calcium [30], each cell line was also co-transfected with pcDNA3.1 plasmid containing jelly fish aequorin cDNA. Twenty four hours before the assay, cells were seeded in 96-well plates (Corning, Inc) and TRP channel expression was induced with 0.5 μg/ml tetracycline. On the day of the assay, culture media was removed and cells were incubated with assay buffer (F12 containing 30 mM HEPES for TRPA1, TRPM8, TRPV1 and TRPV3; F12 containing 30 mM HEPES, 1 mM CaCl_2_, and 0.3% BSA for TRPV4) containing 15 μM coelenterazine (P.J.K, Germany) for 2 hours. Antagonists were added for 2.5 min prior to addition of an agonist. The luminescence was measured by a CCD camera based FLASH-luminometer built by Amgen, Inc. The following agonists were used to activate TRP channels: 80 μM AITC for TRPA1, 1 μM icilin for TRPM8, 0.5 μM capsaicin for TRPV1, 200 μM 2-APB for TRPV3, and 1 μM 4α-PDD for TRPV4. Luminescence signal was captured for 1 min after compound addition and for 1 min after the agonist addition, thus generating agonism and antagonism data for each compound from the same assay. Compound activity was calculated using GraphPad Prism 4.01 (GraphPad Software Inc, San Diego, CA).

### Cold antagonism assay (^45^Ca^2+ ^uptake)

Twenty four hours before the assay, cells were seeded in 96-well plates (Amersham) and TRPA1 expression was induced with 0.5 μg/ml tetracycline. Cold activation of TRPA1 was followed as a function of cellular uptake of radioactive calcium (^45^Ca^2+^, Perkin Elmer). All the ^45^Ca^2+ ^uptake assays had a final ^45^Ca^2+ ^concentration of 10 μCi/ml. The Cold antagonist assay was performed as follows: Compounds were pre-incubated for 1 min at room temperature with CHO cells expressing TRPA1 in F-12 medium supplemented with BSA, 0.1 mg/ml, and 15 mM HEPES. ^45^Ca^2+ ^was added in F-12 and the plates were left on a chilling plate unit (Torrey Pines ECHO Therm) for 12 min allowing the final temperature of 3.5°C to be reached. Plates were washed twice with phosphate-buffered saline containing 0.1 mg/ml BSA. Radioactivity was measured using a MicroBeta Jet (Perkin-Elmer Inc). Data were analyzed using GraphPad Prism 4.01 (GraphPad Software Inc., San Diego, CA). Maximum ^45^Ca^2+ ^uptake in response to cold was considered as 100% in calculating the IC_50 _values.

### Electrophysiology

CHO cells expressing hTRPA1 or rTRPA1 were studied by whole-cell voltage-clamp recording using an automated PatchXpress 7000A workstation (Axon Instruments, now part of Molecular Devices). The cells were grown in T-75 flasks with ~70% confluence and the hTRPA1 or rTRPA1 were induced for 24 hours with 0.5 ug/ml tetracycline prior experiment. Cell suspensions were prepared with normal splitting procedure with the use of 0.05% Trypsin-EDTA. The cells were allowed to recover in culture media at 37°C for half an hour before they were re-suspended in extracellular recording solution for final recording. The extracellular recording solution contained (in mM): 62.5 CsCl, 75 CsF, 2.5 MgCl_2_, 10 HEPES, 5 EGTA, pH 7.2. The intracellular recording solution contained (in mM): 140 NaCl, 5 KCl, 2 CaCl_2_, 1.1 MgCl_2_, 10 HEPES, 11 Glucose, pH 7.4. Experiments were performed at a holding potential of -70 mV with a predefined voltage-clamp protocol. Once the protocol was triggered the PatchXpress automatically load the cells and add the test compounds in designed sequence. Under this experimental condition the activation of hTRPA1 or rTRPA1 produced inward current. AITC (80 uM) was used to serve as a control agonist for both hTRPA1 and rTRPA1 channels. The data were recorded on a computer hard disk and the analysis was performed off line with Clampfit software (Axon Instruments, now part of Molecular Devices).

## Abbreviations

AITC: allylisothiocyanate; 

TRPA1: transient receptor potential ankyrin 1; 

TRPM8: transient receptor potential melastatin 8; 

TRPV1: transient receptor potential vanilloid type 1; 

TRPV3: transient receptor potential vanilloid type 3; 

TRPV4: transient receptor potential vanilloid type 4; 

2-APB: 2-Aminoethoxydiphenyl borate; 

4αPDD: 4alpha-Phorbol 12,13-didecanoate; 

CHO: Chinese hamster ovary; 

AMG2504: 4-nitro-N-(2,2,2-trichloro-1-((4-chlorophenyl)sulfanyl)ethyl)benzamide; 

AMG5445: 4-methoxy-N-(2,2,2-trichloro-1-((4-chlorophenyl)sulfanyl)ethyl)benzamide; 

AMG7160: 4-bromo-N-(2,2,2-trichloro-1-((4-chlorophenyl)sulfanyl)ethyl)benzamide; 

AMG9090: N-(2,2,2-trichloro-1-((4-chlorophenyl)sulfanyl)ethyl)benzamide.

## Competing interests

All authors are employed by for profit company, Amgen, Inc.

## Authors' contributions

LK carried out the FLASH luminomoter assays, RT conducted ^45^Ca^2+ ^uptake assays, BG carried out electrophysiology assays, and WW conducted selectivity assays. LK, RT, BG and WW wrote methods. DCI advised and supervised electrophysiology assays. NN prepared the compounds and contributed to writing of results and discussion. NRG conceived of the study, participated in its design and coordination. LK and NRG wrote the manuscript. All authors read and approved the final manuscript.
